# Integrated Spectral Sensitivity as Physics-Based Figure of Merit for Spectral Transducers in Optical Sensing

**DOI:** 10.3390/s25020440

**Published:** 2025-01-13

**Authors:** Felix L. McCluskey, Anne van Klinken, Andrea Fiore

**Affiliations:** Department of Applied Physics and Science Education, Eindhoven Hendrik Casimir Institute, Eindhoven University of Technology, P.O. Box 513, 5600 MB Eindhoven, The Netherlands

**Keywords:** optical sensor, biosensors, figure of merit, Cramér–Rao lower bound, photonic crystal, Fabry–Pérot cavity, sensing performance, sensitivity

## Abstract

The design of optical sensors aims at providing, among other things, the highest precision in the determination of the target measurand. Many sensor systems rely on a spectral transducer to map changes in the measurand into spectral shifts of a resonance peak in the reflection or transmission spectrum, which is measured by a readout device (e.g., a spectrometer). For these spectral transducers, figures of merit have been defined which are based on specific assumptions on the readout and the data analysis. In reality, however, different transducers achieve optimal performance with different types of readout. Additionally, some transducers present a more complex spectral response for which existing figures of merit do not apply. In this paper, we investigate an approach to quantifying the potential performance of a given transducer for a more general class of readout methods. Starting from the Cramér–Rao lower bound, we define a new figure of merit, the integrated spectral sensitivity, which is directly related to the physical limit of precision and applicable to a wide variety of sensing systems. We apply this analysis to two different examples of transducers. The results bring useful insights into the design of optical sensors.

## 1. Introduction

**Figure 1:**
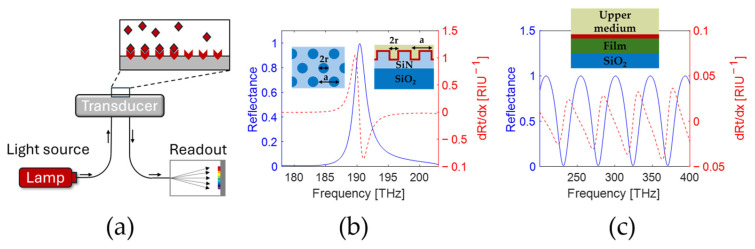
(**a**) Schematic representation of the sensing system. Reflectance spectrum (blue) of (**b**) a photonic crystal (insets with biolayer in red) and (**c**) a Si/SiO_2_ multilayer (inset with biolayer in red) and its derivative with respect to the measurand (red).

Optical sensing systems can be broadly divided into two categories. In the first case, the property to be measured is directly imprinted onto the light transmitted and reflected, scattered or emitted by the object under study—that is, for example, the situation in imaging or spectroscopy problems. In a second category of applications, a transducer is used to map a physical, chemical or biological parameter of interest into a change of (mostly) transmittance or reflectance, which is then probed by a light beam. In this second case, which includes, for example, most biosensors and fiber sensors, the sensor system is constituted by the transducer, the propagation channel (typically fiber or free space) and a readout unit including a light source and one or more photodetectors (see Figure 1a). For example, for label-free optical biosensors, the transducer can be based on a dielectric or dielectric/metal structure which creates a nanophotonic resonance, and the readout unit may consist of a combination of laser and camera [1] for spatial transduction, or a broad light source and a spectrometer [2,3] for spectral transduction. The latter, where changes in the measurand cause changes in the spectrum, offers several advantages. Firstly, the spectrum (in contrast to the power or phase) is relatively stable during propagation, also via fibers. Secondly, the transmittance or reflectance spectrum is relatively easy to measure, e.g., using a spectrally broad light source and spectrally resolved detection (e.g., with a spectrometer) or by tuning a narrow light source and measuring the total transmitted/reflected power. Examples include Fiber Bragg gratings (FBGs) [4], photonic crystals (PhCs) [5] and nanoplasmonic sensors [6].

It is obvious that the combination of transducer and readout determines the overall sensing performance and the cost of the system, where typically, the readout represents the largest fraction of the equipment cost. As commercial spectral readout systems mostly consist of general-purpose high-resolution instruments such as spectrometers and tunable lasers, research efforts have focused on the optimization of spectral transducers in combination with high-resolution readouts. The most commonly used figure of merit (FoM) for spectral transducers, $FoM_{\lambda }=S_{\lambda }/\Delta \lambda_{FWHM}$, is defined as the ratio of wavelength sensitivity and linewidth. Here, the wavelength sensitivity $S_{\lambda }=∆\lambda /∆x$ refers to the shift ∆λ of a spectral feature, usually a peak or a dip, originating from a change ∆x in the measurand, while $∆\lambda_{FWHM}$ indicates the linewidth of the feature. This FoM reflects the role of the linewidth in the precision of the wavelength determination from a measurement of the spectrum, as pointed out in many studies [7,8]. Recently, adaptations to this FoM have been proposed which take into account the depth of the modulation [9,10] and other aspects of the sensing system [11]. The choice of an FoM should of course be based on its relation to the imprecision achievable with a given transducer and readout system. This relation has so far been investigated based on numerical or experimental spectral data and processing the available data to determine the wavelength of the spectral feature, such as peak tracking [7], curve fitting [8], or the centroid method [11]. This leaves open the question whether the observed imprecisions represent the real physical limit for that particular system or may be limited by the choice of data processing. This is particularly critical when the transducer presents a complex spectrum with several peaks or other features, as is the case in many nanophotonic structures. Additionally, the justification for all proposed FoMs is based on the assumption of a high-resolution spectral readout, and in particular, the dependence of the wavelength imprecision on $\Delta \lambda_{FWHM}$ has been investigated only in combination with a high-resolution readout [7,8]. However, in view of the cost and lack of robustness of high-resolution spectral instrumentation, it is worthwhile exploring low-cost spectral readout methods. For example, for the readout of label-free biosensors, LED arrays have been used to replace spectrometers [12], and spectral–spatial mapping in combination with cameras has been proposed [13]. Recently, our group introduced a different multispectral readout method in which a broad light source is used in combination with an array of resonant-cavity photodetectors [14]. We have shown theoretically and experimentally that this simple method allows determination of the resonant wavelength of a transducer with a precision exceeding that provided by high-resolution instrumentation. As both the physical readout mechanism and the subsequent data processing are different in these alternative readout methods, the $FoM_{\lambda }$ and other FoMs proposed so far are not expected to apply to the corresponding transducers. More generally, there is a need for more general FoMs which can be applied to a broad class of readout methods, preferably without making any assumption about the choice of data processing. We can also ask whether it is possible to quantify the ultimate sensing performance of a transducer, i.e., the one obtained with an “ideal” readout optimized for that particular transducer. This would allow comparing different transducers independently of readout and data processing.

This work addresses the definition of FoMs by starting from the expression for the Cramér–Rao lower bound (CRLB) for the imprecision of a sensing system based on spectral transduction. This leads to the definition of a simple FoM, without any assumption about the data analysis method. From its analytical expression, we derive important consequences on the choice of transducer–readout combinations, which we verify numerically for two widely used classes of transducer responses, namely, a single-resonance spectrum (such as a PhC resonance) and a multipeaked response (e.g., from a Fabry–Pérot). We then analyze two types of readouts, the usual “spectrometric” readout, and the “ideal” readout (i.e., the best possible readout for that particular transducer). For both cases, simple expressions for the FoM are derived which quantify the ultimate performance of a transducer for the corresponding class of readout. We then use them to study the impact of the transducer’s design parameters on its sensing performance and compare the potential of different types of transducers for a particular case of label-free biosensing, showing that the proposed approach brings new insights and points to new directions in transducer design. While we focus our analysis on two specific examples, it is applicable to many other spectral transducers, such as the commonly used localized surface plasmon resonance (LSPR) sensors—the only differences are found in the reflectance spectrum and the sensitivity.

## 2. Materials and Methods

While the approach outlined below is very general and applicable to any optical sensing system based on spectral transduction, this work will only refer to the case of label-free biosensing to provide specific examples. In this case, the measurand of interest x is the refractive index at the surface of the transducer (commonly referred to as $n_{b}$), and the sensing goal is the measurement of small changes dx due to the selective binding of biomolecules [15]. It is assumed that these refractive index changes occur in a layer of 10 nm thickness. Only two examples of transducers are considered, namely, a narrow-linewidth guided-mode resonance formed by a Si_3_N_4_ PhC, and a silica Si/SiO_2_ multilayer producing multiple Fabry-Pérot resonances with low finesse (see Figure 1). Both types of transducers were previously used for biosensing [16,17,18], and they are chosen in view of the very different characteristics of their reflection spectrum to highlight the generality of our approach and proposed FoM. Figure 1b shows the reflectance spectra for a hexagonal PhC (for a SiN thickness = 410 nm, lattice constant = 1140 nm, hole radius = 433.2 nm), calculated using a commercial finite-element solver (COMSOL Multiphysics). Figure 1c shows the reflectance spectrum for a 930 nm thick Si layer on silica, calculated using the transfer-matrix method. The PhC design of [19] has been used in view of its polarization-degenerate mode, adapting several parameters to tune the resonance around 1550 nm (193 THz) using a different material platform. This choice of material and thickness for the multilayer structure provides a strong reflectance modulation and produces a sufficient number of fringes in the frequency range of interest, while the exact value of the thickness will not affect the outcome of the results. In both cases, water is assumed as the top medium, with a refractive index of 1.32. For simplicity, all materials are assumed to be dispersionless in the investigated frequency range for both transducers. The sensitivity of the reflectance spectrum to index changes in the 10 nm thick layer above the transducer surface, $\partial R_{t}/\partial x$, is also shown in Figure 1b,c (dashed line) for both cases. As expected, the main effect in both cases is a red-shift of the resonant peaks, quantified by a surface sensitivity (in frequency) dν/dx of 1.829 THz/RIU for the PhC and 0.852 THz/RIU for the multilayer, determined for the central peak in Figure 1c. For the calculation of the surface sensitivity, the frequency shift was determined from a Lorentzian fit of the response peak for the PhC. For the multilayer structure, the frequency shift is calculated from a linear fit for the two points with the largest slope around the central peak and averaged.

Figure 1b,c clearly show a well-isolated peak for the PhC response, while for the multilayer, the free spectral range is comparable to the linewidth. It can, therefore, be expected that different readout schemes and data analysis approaches (e.g., curve fitting methods) will be optimal for the two transducers. Also, more complex transducers (e.g., multilayers with more layers) can have more complex spectra which are not defined by a single peak. In the following, an analytical framework is provided which allows quantitative comparison of the potential sensing performance of these and other transducers, independently of the shape of their spectrum.

## 3. Results

### 3.1. Integrated Spectral Sensitivity as Physics-Based FoM

In order to derive a general FoM for a broader class of sensors and readout methods, several assumptions need to be made. In the following, we will focus on the case of a spectrally broad light source and an array of photodetectors with spectral selectivity (readout channels) (Figure 1a), which is especially relevant for low-cost readout systems. The case of a light source with very small linewidth which is swept through the spectrum (as in the case of readout system based on a tunable laser) is treated in Appendix A. We consider a spectrally broad light source (for example, a halogen lamp or a light-emitting diode) emitting in a frequency range between $\nu_{min}$ and $\nu_{max}$. The optical power density within this spectral range is assumed for simplicity to be uniform, $P_{\nu }\left(\nu \right)=P_{\nu }$, leading to the total optical power $P=P_{\nu }∆\nu$. We note that the assumption of a flat spectral density is not crucial; however, the source spectrum must be known and constant in time to achieve high sensing precision. It is assumed that the width of the spectral range $∆\nu =\nu_{max}-\nu_{min}$ is much smaller than the mean frequency ($∆\nu \ll \bar{\nu }=(\nu_{max}+\nu_{min})/2$), so that the responsivity of the readout channel can be approximated as $R_{i}\left(\nu \right)=\frac{e}{h\bar{\nu }}T_{i}\left(\nu \right),\;$where $T_{i}\left(\nu \right)$ is the transmission of the spectral filter associated with that channel (including all loss in the optical system and in the photodetector). In practice, $T_{i}\left(\nu \right)$ is defined, for example, by the geometry and the grating in a grating spectrometer, or by the filter integrated with the photodetectors in the multispectral readout case [14]. The photocurrent $I_{i}$ in each readout channel is then given by

(1) $$I_{i}=P_{\nu }\int_{\nu_{min}}^{\nu_{max}}R_{t}\left(\nu \right)R_{i}\left(\nu \right)d\nu \;≅\;P_{\nu }\frac{e}{h\bar{\nu }}\int_{\nu_{min}}^{\nu_{max}}R_{t}\left(\nu \right)T_{i}\left(\nu \right)d\nu .$$

The measurand of interest, x, influences only the transducer’s reflection spectrum $R_{t}\left(\nu \right)$, so the sensitivity of this photocurrent on the measurand is given by

(2) $$\frac{\partial I_{i}}{\partial x}=P_{\nu }\frac{e}{h\bar{\nu }}\int_{\nu_{min}}^{\nu_{max}}\frac{\partial R_{t}\left(\nu \right)}{\partial x}T_{i}\left(\nu \right)d\nu .$$

In order to differentiate the effect of the measurand from fluctuations in the light source power, it is essential that the transducer’s reflection spectrum shows an anticorrelated spectral change upon a change in x. We conclude that the derivative $\partial R_{t}\left(\nu \right)/\partial x$ must change sign within the spectral range ∆ν, containing minima with $\partial R_{t}\left(\nu \right)/\partial x<0$ and maxima with $\partial R_{t}\left(\nu \right)/\partial x>0$. In the following, $∆\nu_{t}$ will be used to describe the typical frequency difference between these minima and maxima of $\partial R_{t}\left(\nu \right)/\partial x$ within the spectral range ∆ν, i.e., the width of the spectral features. For a Lorentzian line shape, this corresponds to the full-width at half-maximum (FWHM). Our more general definition of $∆\nu_{t}$ also allows description of the typical spectral width in the case of a complex reflection spectrum other than a single peak or dip.

According to information theory, based on the Fisher information matrix [20], the fundamental limit to imprecision, at which the measurand x can be determined from the photocurrents $I_{i}$ of the n photodetectors, is given by the Cramér–Rao lower bound (CRLB). More specifically, under very general conditions, the CRLB represents a lower limit to the standard deviation of an unbiased estimator of x. Assuming that the noise currents on the readout photodetectors follow a Gaussian distribution with an equal standard deviation $\sigma_{I}$ and are statistically independent, the CRLB is given by

(3) $$CRLB=\frac{\sigma_{I}}{\sqrt{\sum_{i=1}^{n}{\left(\frac{\partial I_{i}}{\partial x}\right)}^{2}}}=\frac{\sigma_{I}}{P_{\nu }∆\nu \frac{e}{h\bar{\nu }}\;\frac{1}{∆\nu }\sqrt{\sum_{i=1}^{n}{\left(\int_{\nu_{min}}^{\nu_{max}}\frac{\partial R_{t}\left(\nu \right)}{\partial x}T_{i}\left(\nu \right)d\nu \right)}^{2}}}=\frac{\sigma_{I}}{P\frac{e}{h\bar{\nu }}S_{int}}.$$

Here, the “integrated spectral sensitivity” $S_{int}$ is defined as

(4) $$S_{int}\equiv \frac{1}{∆\nu }\sqrt{\sum_{i=1}^{n}{\left(\int_{\nu_{min}}^{\nu_{max}}\frac{\partial R_{t}\left(\nu \right)}{\partial x}T_{i}\left(\nu \right)d\nu \right)}^{2}},$$

which describes the collective sensitivity of all readout photocurrents to changes in the measurand x. The integrated spectral sensitivity $S_{int}$ has the unit of $x^{-1}$ and depends both on the properties of the transducer, due to the term $\partial R_{t}\left(\nu \right)/\partial x$, and the properties of the readout, described by ∆ν and $T_{i}\left(\nu \right)$. One can note that the term $P\frac{e}{h\bar{\nu }}/\sigma_{I}$ in Equation (3) equals the “available” signal-to-noise ratio (SNR) in the sensing system (corresponding to the maximum photocurrent that can be generated given the power of the light source), so Equation (3) can also be written as $CRLB=\frac{1}{SNR}\;\cdot \;\frac{1}{S_{int}}$. The SNR is determined by the power of the light source and the noise performance of the photodetectors, while $S_{int}$ depends on the spectral characteristics of the transducer and readout. For this reason, the integrated spectral sensitivity $S_{int}$ represents a general, physics-based FoM for any sensing system based on spectral transduction, provided the assumptions about the light source and photodetectors are satisfied. In particular, it does not rely on any assumption on the algorithm (peak fitting or other) used to predict the parameter x from the spectrum. It should be noted that the assumption of Gaussian and statistical independent noise currents in the photodetectors is very general and applies to thermal-noise-limited and shot-noise-limited detection (for the latter in the limit of large photon number).

While this derivation was based on the assumption of a spectrally broad light source, similar expressions for the CRLB and the integrated spectral sensitivity can be derived for the case of a light source with narrow linewidth, such as laser-based readout systems, as is described in more detail in Appendix A.

We now want to compare the integrated spectral sensitivity $S_{int}$ for different transducers. To this aim, more specific assumptions are made about the characteristics of the readout.

### 3.2. Spectrometric Readout

Many readout schemes rely on the separation of spectral information into different closely spaced frequency bands, either in space (e.g., grating-based spectrometers or multispectral arrays [14]) or time (e.g., sequential measurement using a tunable filter). Regardless of the technical realization, the frequency-dependent response of the n readout channels can be approximately described by a Lorentzian line shape with linewidth δν (full-width half maximum) using

(5) $$T_{i}\left(\nu \right)=\frac{\pi \;\delta \nu }{2K}L\left(\nu -v_{i}\right).$$

**Figure 2:**
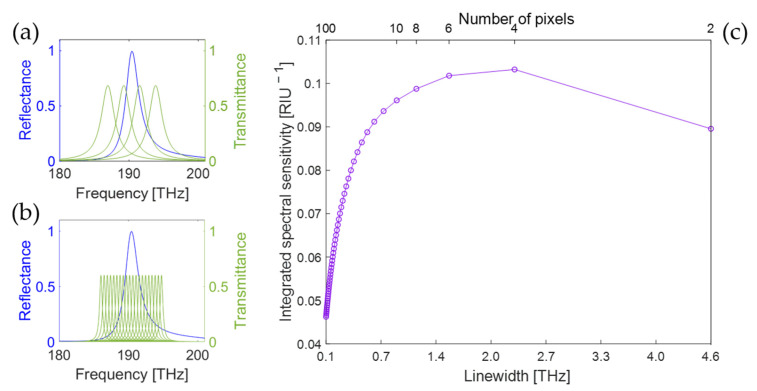
(**a**,**b**) Reflectance spectrum of PhC transducer (blue line) with the transmission functions for the readout channels of a spectrometric readout (green lines) for (**a**) n=4 and (**b**) n=20. (**c**) Calculated integrated spectral sensitivity for PhC sensor with spectrometric readout of various linewidths.

Here, $L\left(\nu \right)=\frac{1}{\pi }\;\frac{\delta \nu /2}{\nu^{2}+{\left(\delta \nu /2\right)}^{2}}$ is a Lorentzian line shape centered around ν=0, which is integral normalized to $\int_{-\infty }^{\infty }L\left(\nu \right)d\nu =1$. The linewidth is fixed as $\delta \nu \equiv \frac{∆\nu }{n}$ to be equal to the spectral range ∆ν divided by the number n of readout channels and equal to the channel spacing, $\delta \nu =\nu_{i}-\nu_{i-1}$. Considering specifically the more common case of spatially distributed readout channels (e.g., grating with photodetector array or array of filters and photodetectors), the readout channels together cannot absorb more than 100% of the incoming light at a certain frequency. To keep the maximum of their sum normalized to 1, the transmission functions $T_{i}\left(\nu \right)$ are, therefore, scaled by a factor $K=\sum_{j=-\infty }^{\infty }\frac{1}{4j^{2}+1}\;\approx 1.48$. An example for four readout channels is shown in Figure 2a. Importantly, the best possible physical realization of a spectrometric readout is assumed, where the optical throughput does not depend on the number or linewidth of the spectral channels. This is not necessarily the case in practice—for example, in a grating spectrometer, changing the resolution requires reducing the size of the input slit, which reduces the optical throughput, while in a multispectral array, the throughput scales as 1/n. However, here we are interested in the physical limits achievable with a spectrometric readout and not in the specific implementations.

Substituting the expression from Equation (5) into Equation (4), the following is obtained

(6) $$S_{int}=\frac{1}{∆\nu }\sqrt{\sum_{i=1}^{n}{\left(\frac{\pi \;\delta \nu }{2K}\int_{\nu_{min}}^{\nu_{max}}\frac{\partial R_{t}\left(\nu \right)}{\partial x}L\left(\nu -v_{i}\right)d\nu \right)}^{2}}=\frac{\pi \;\delta \nu }{2K∆\nu }\sqrt{\sum_{i=1}^{n}{\left({\left.\frac{\partial R_{t}\left(\nu \right)}{\partial x}\times L\left(\nu \right)\right|}_{v_{i}}\right)}^{2}}.$$

In order to draw conclusions from this expression, two regimes for the readout linewidth, $\delta \nu \ll ∆\nu_{t}$ and $\delta \nu \gg ∆\nu_{t}$, are treated separately.

In the limit of small readout linewidth ($\delta \nu \ll ∆\nu_{t}$), the sum over the readout channels can be considered as a summation over n closely spaced frequency bands of width δν, as their central frequencies $v_{i}$ are spaced by their linewidth δν. In the limit of small linewidth δν, this sum with n=∆ν/δν summands can be approximated by an integral over the frequency range ∆ν:

(7) $$S_{int,HR}=\frac{\pi \;\delta \nu }{2K∆\nu }\sqrt{\frac{1}{\delta \nu }\int_{\nu_{min}}^{\nu_{max}}{\left({\left.\frac{\partial R_{t}\left(\nu \right)}{\partial x}\times L\left(\nu \right)\right|}_{v^{\prime }}\right)}^{2}d\nu^{\prime }}.$$

For $\delta \nu \ll ∆\nu_{t}$, the convolution is well approximated by ${\left.\frac{\partial R_{t}\left(\nu \right)}{\partial x}\times L\left(\nu \right)\right|}_{v^{\prime }}\approx \frac{\partial R_{t}}{\partial x}\left(\nu^{\prime }\right)$, leading to

(8) $$S_{int,HR}=\frac{\pi }{2K∆\nu }\sqrt{\delta \nu \int_{\nu_{min}}^{\nu_{max}}{\left|\frac{\partial R_{t}}{\partial x}\left(\nu^{\prime }\right)\right|}^{2}d\nu^{\prime }}$$

This can be rewritten as a function of the number of spectral points n as

(9) $$S_{int,HR}=\frac{\pi }{2K\sqrt{n}}\sqrt{\frac{1}{∆\nu }\int_{\nu_{min}}^{\nu_{max}}{\left|\frac{\partial R_{t}}{\partial x}\left(\nu^{\prime }\right)\right|}^{2}d\nu^{\prime }}.$$

For a specific choice of transducer, the spectral sensitivity shows a $S_{int,HR}\sim \sqrt{\delta \nu }$ behavior (or equivalently, $S_{int,HR}\sim 1/\sqrt{n}$) in the regime of small linewidth δν). For the assumptions that were made for the case of a spectrometric readout, the small linewidth regime represents a typical multi-purpose high-resolution readout scheme, such as a grating-based spectrometer (δν = bin width). The relation $S_{int,HR}\;\sim \sqrt{\delta \nu }$, shows that for any type of transducer, the sensing performance degrades when the readout linewidth is decreased well below the width of the features in the transducer reflectance (or equivalently, when the number of spectral points n is increased). Consequently, a high-resolution readout unit hampers the achievable imprecision in this case. This conclusion is opposite to that of [8], which was based on a Montecarlo analysis and did not consider, to the best of our understanding, the effect of δν on the signal level and, therefore, on the signal-to-noise ratio. It is, in fact, intuitive that in the limit δν→0, the photocurrents tend to zero and no sensing information is available.

In the case of a large readout linewidth ($\delta \nu \gg ∆\nu_{t}$), there is only a small number n of readout channels, for which we sum the respective contribution. For each of these readout channels, the integral in Equation (6) includes positive and negative contributions of $\partial R_{t}\left(\nu \right)/\partial x,$ as their typical feature width $∆\nu_{t}$ is much smaller than the linewidth δν. This results in a degraded spectral sensitivity, which is unfavorable for the performance of the sensing system.

From the above considerations, we conclude that the integrated spectral sensitivity is expected to degrade both in the case of very small and in very large readout linewidth δν. It is, therefore, expected that the maximum sensitivity, and thereby the optimum performance of the sensing system, is achieved if the readout linewidth δν and the typical feature width $∆\nu_{t}\;$in $\partial R_{t}\left(\nu \right)/\partial x$ are of the same order.

To verify the predicted behavior of the spectral sensitivity, the two cases shown in Figure 1 were numerically studied: a PhC transducer featuring a single peak in the reflection spectrum, and a multilayer stack with equally spaced Fabry–Pérot fringes. $S_{int}$ is systematically calculated for readout channels with different linewidth, and the observed trends are compared to the analytical derivation.

The PhC transducer in Figure 1b presents a single peak in the reflectance spectrum at 190.5 THz, with linewidth $∆\nu_{S}=\;$2.3 THz. Readout schemes in the frequency range ∆ν from 186 THz to 195 THz, which are symmetric around the transducer reflectance peak, are considered. To compare the expected sensing performance of spectrometric readout schemes with different linewidth δν, the integrated spectral sensitivity $S_{int}\;$from Equation (4) is numerically calculated as an FoM. In Figure 2, two examples of the transmission functions $T_{i}\left(\nu \right)$ for n=4 (Figure 2a) and n=20 (Figure 2b) are shown together with the transducer reflectance for illustration. In Figure 2c, the dependence of $S_{int}\;$on the linewidth δν is plotted for even numbers n of readout channels. As derived analytically, the integrated spectral sensitivity rapidly decreases for small linewidths $\delta \nu \ll ∆\nu_{t}$. It can be noted that in most practical spectrometric systems, the optical throughput decreases for smaller linewidths, resulting in a steeper decrease in the integrated spectral sensitivity with respect to Figure 2c. The graph shows a maximum in spectral sensitivity for the case n=4 with the linewidth δν=2.3 THz, which matches the linewidth of the peak in the transducer reflectance. For a reflectance spectrum featuring a single Lorentzian peak, it can be shown [14] that the readout performs optimally for a linewidth δν equal to the linewith of the transducer reflectance peak. A possible implementation of a four-pixel multispectral readout using an array of resonant-cavity detectors, a suitable data processing method and an experimental proof that this type of low-resolution spectrometric readout can provide better performance than a high-resolution one were also reported in [14].

**Figure 3:**
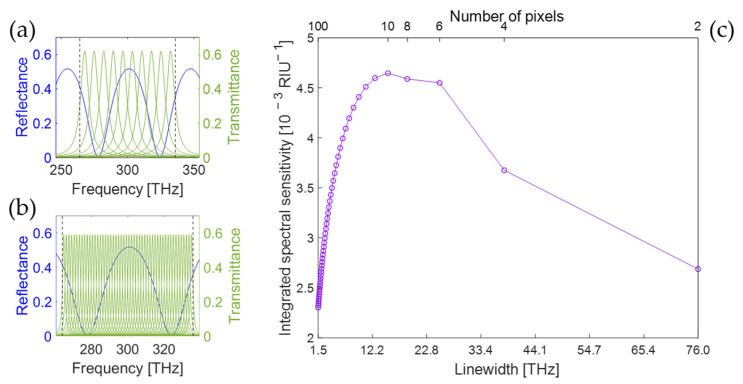
(**a**,**b**) Reflectance spectrum of multilayer stack (blue line) with the transmission functions for the readout channels of a spectrometric readout (green lines) for (**a**) n=10 and (**b**) n=50. (**c**) Calculated integrated spectral sensitivity for multilayer stack with spectrometric readout of various linewidths.

The Si/SiO_2_ multilayer structure in Figure 1c shows the typical reflection spectrum of a Fabry–Pérot cavity, with a periodicity of 46 THz. The dips are relatively broad due to the limited reflectance of the two interfaces. As described above, the typical frequency difference between maxima and minima in $\partial R_{t}\left(\nu \right)/\partial x$ is given by $∆\nu_{t}$ = 14 THz. The frequency range ∆ν is chosen from 246 THz to 336 THz, which includes about 1.5 periods of the FP reflectance spectrum. Again, $S_{int}$ calculated from Equation (4) is used as an FoM to compare the expected sensing performance for various spectrometric readout schemes with different linewidth δν. In Figure 3, two examples of transmission functions $T_{i}\left(\nu \right)$ for n=10 (Figure 3a) and n=50 (Figure 3b) are shown for illustration. In Figure 3c, the dependence of the integrated spectral sensitivity $S_{int}$ on the linewidth is shown. Again, we observe a significant decrease in integrated spectral sensitivity towards small linewidths $\delta \nu \ll ∆\nu_{t}$. For linewidths around $∆\nu_{t}$ and above, the behavior is more complex and depends on the exact choice of the wavelength range ∆ν. Indeed, while for small linewidths, the whole frequency range is evenly covered with readout channels, for large linewidths, the contribution of the few readout channels to the spectral sensitivity largely depends on the relative position to the FP fringes, making the performance much more dependent on the definition of the frequency range ∆ν. Regardless of this choice, a global maximum of the spectral sensitivity is observed for δν≈ 16 THz (for n≈10), which is comparable to $∆\nu_{t}$ = 14 THz. Also by this calculation, we confirm the analytical conclusion that the best sensing performance is expected for a readout scheme with a linewidth of the same order as the transducer’s reflectance features.

Considering the role of the readout linewidth, we conclude that the simulation results support our analytical argumentation that the sensing performance is optimized by using readout channels with a linewidth of the same order as the typical feature width in the reflectance spectrum of the transducer. This underlines our conclusion that the use of high-resolution readout equipment does not always provide the best possible performance and biases the development of optimized transducers towards small linewidths. In contrast to the common approach of relying on $FoM_{\lambda }$ for the transducer alone, using the integrated spectral sensitivity as an FoM allows comparing the collective performance of a transducer and a readout unit, which evidently need to be co-optimized.

In order to explore the ultimate sensing potential of a given transducer, we now consider the theoretical limit to the integrated spectral sensitivity that can be reached under ideal readout conditions.

### 3.3. Ideal Readout

**Figure 4:**
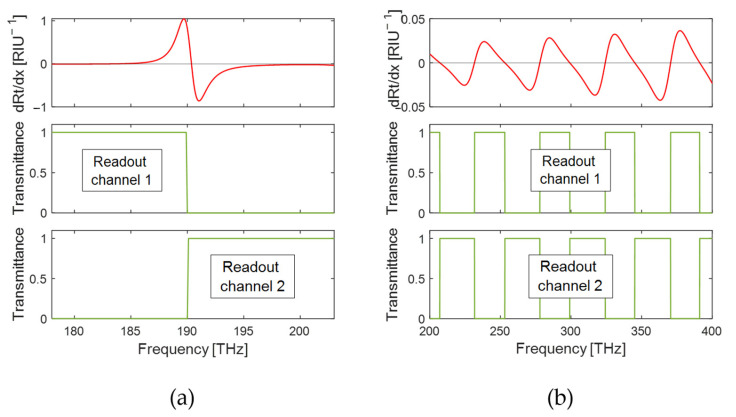
Reflectance spectrum of (**a**) the PhC transducer and (**b**) a multilayer transducer, together with the two respective transmission functions for the ideal readout.

Starting from the definition of the spectral sensitivity in Equation (4), a hypothetical set of transmission functions $T_{i}\left(\nu \right)$ are assumed, which maximize $S_{int}$ for a specific transducer with reflectance derivative $\partial R_{t}\left(\nu \right)/\partial x$. As $\partial R_{t}\left(\nu \right)/\partial x$ changes sign within the integration range, for transmission functions with the physical constraint ${0\leq T}_{i}\left(\nu \right)\leq 1$, at least two readout channels are required to maximize the sum of the squared integrals. Then for each readout channel, the square of the integral is maximized if the product has the same sign along the whole integration range. As the sum of the transmission functions cannot exceed 1 at any frequency, the sum of the squared integrals is maximized if all positive values of $\partial R_{t}\left(\nu \right)/\partial x$ are included in the integral for one readout channel, and if all negative values of $\partial R_{t}\left(\nu \right)/\partial x$ are included for another readout channel. This is the case for two readout channels with transmission functions:

(10) $$T_{1,2}\left(\nu \right)=\frac{1}{2}\left(1\pm sgn\left(\frac{\partial R_{t}\left(\nu \right)}{\partial x}\right)\right),$$

which are defined within the spectral range ∆ν. In Figure 4, these transmission functions are plotted for the PhC and the multilayer transducers of Figure 1 for illustration. While the discontinuous behavior of the transmission function in each zero-crossing of $\partial R_{t}\left(\nu \right)/\partial x$ is clearly unphysical, this choice represents the theoretical limit to the spectral sensitivity, which cannot be exceeded by any physical readout scheme. Substituting these ideal characteristics from Equation (10) into Equation (4) yields

(11) $$S_{ideal}=\frac{1}{∆\nu }\frac{1}{2}\sqrt{{\left(\int_{\nu_{min}}^{\nu_{max}}\frac{\partial R_{t}\left(\nu \right)}{\partial x}\left(1+sgn\left(\frac{\partial R_{t}\left(\nu \right)}{\partial x}\right)\right)d\nu \right)}^{2}+{\left(\int_{\nu_{min}}^{\nu_{max}}\frac{\partial R_{t}\left(\nu \right)}{\partial x}\left(1-sgn\left(\frac{\partial R_{t}\left(\nu \right)}{\partial x}\right)\right)d\nu \right)}^{2}}.$$

In the common case where $R_{t}\left(\nu \right)$ consists of one or multiple resonances which shift in frequency, $\partial R_{t}\left(\nu \right)/\partial x$ has equal positive and negative values, and its integral over frequency is approximately zero: $\int_{\nu_{min}}^{\nu_{max}}\partial R_{t}\left(\nu \right)/\partial x\;d\nu ≅0$. Rewriting the expression $\partial R_{t}\left(\nu \right)/\partial x\;sgn\left(\partial R_{t}\left(\nu \right)/\partial x\right)=\left|\partial R_{t}\left(\nu \right)/\partial x\right|$ leads to

(12) $$S_{ideal}≅\frac{1}{∆\nu }\frac{1}{\sqrt{2}}\int_{\nu_{min}}^{\nu_{max}}\left|\frac{\partial R_{t}\left(\nu \right)}{\partial x}\right|d\nu \;.$$

This expression for the ideal spectral sensitivity, valid for shifting resonances and ideal readout, quantifies the spectral response of the transducer throughout the frequency range of interest. As such, it can be used as a general indicator for the possible performance of a transducer and allows a fair comparison across a large class of transducers. It can be noted that the ideal readout is not only a theoretical tool: it is in practice possible to realize filters whose transmission approximates a target spectral function. The optimization of optical filters for specific sensing goals has indeed already been demonstrated in the context of multivariate optical computing [21], and such filters are available commercially [22]. In the simplest implementation, the transmission and reflection channels of a single filter can be used to implement the target spectral functions $T_{1}\left(\nu \right)\;$and $T_{2}\left(\nu \right)=1-T_{1}\left(\nu \right)$.

Comparing Expressions (9) and (12), it is evident that the integrated spectral sensitivity for the ideal readout case is always larger than the one for spectrometric readout. In the cases of the photonic crystal and the multilayered structure, the following values are obtained: $S_{ideal,PhC}=0.27$ RIU^−1^, and $S_{ideal,ML}=0.015$ RIU^−1^. Both values are higher than the maxima in both Figure 2c (0.103 RIU^−1^) and Figure 3c (0.0046 RIU^−1^).

### 3.4. Comparison of Transducers

Having derived simplified expressions for the integrated spectral sensitivity (and therefore, the CRLB) in the two cases of high-resolution spectrometric readout and ideal readout, they can now be applied to assess the performance of given transducers. In particular, the impact of transducer design on the sensing performance can be evaluated, independently of the details of the readout and data processing. As an illustration, this approach is applied to the two transducers in Figure 1, and the results are compared to the traditional figure of merit, $FoM_{\nu }=S_{\nu }/\Delta \nu_{FWHM}=S_{\lambda }/\Delta \lambda_{FWHM}=FoM_{\lambda }$, which has the same unit, but a different meaning than the integrated spectral sensitivity $S_{int}$.

**Figure 5:**
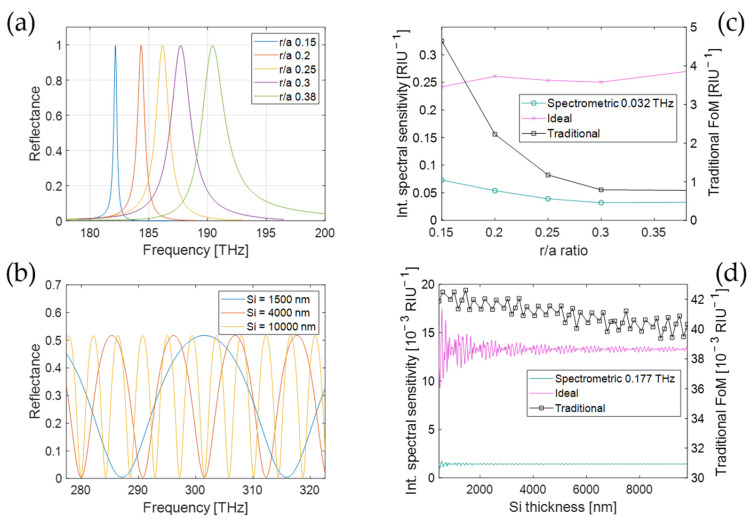
Reflectance spectrum of (**a**) PhC transducer with different normalized radii r/a and (**b**) multilayer structures with different thicknesses. In (**c**,**d**), the corresponding integrated spectral sensitivity for an ideal readout (red crosses, left axis) and a high-resolution spectrometric readout (blue circles, left axis) are shown in comparison with the traditional figure of merit $FoM_{\nu }$ (black squares, right axis).

For the PhC transducer, the investigated design parameter is the normalized radius of the holes (ratio of the hole radius to the lattice constant, r/a). This parameter has an impact on both the linewidth of the reflectance peak and the sensitivity $S_{\nu }=\partial \nu /\partial x$ to a change in the refractive index x within the biolayer, as the electric field intensity on the PhC surface is influenced by the hole size. Here, the normalized radius is varied in the range from r/a=0.15 to r/a=0.38. The corresponding reflectance spectra are shown in Figure 5a. The reflection peaks blue shift from 182.2 THz to 190.5 THz for increasing normalized radius, and the linewidth changes from $∆\nu_{t}=0.32$ THz to $∆\nu_{t}=2.3$ THz. The sensitivities to a refractive index change in the biolayer were found to increase from $S_{\nu }=1.534$ THz/RIU to $S_{\nu }=1.829$ THZ/RIU. The increasing trend with increasing hole radius is consistent with the higher electric field values at the surface of the PhC. For the comparison of the sensing performance of these PhC transducers, a spectral range of ∆ν=8.4 THz is considered, which is centered around the peak frequency for each transducer.

The sensing performance is compared in Figure 5c using three different FoMs: the ideal spectral sensitivity $S_{ideal}$ with ideal readout from Equation (12) (pink crosses); the integrated spectral sensitivity $S_{int,HR}$ with high-resolution spectrometric readout from Equation (8) (for linewidth δν= 0.032 THz, including 256 frequency channels) (green circles); and the traditional figure of merit $FoM_{\nu }=S_{\nu }/\Delta \nu_{FWHM}\;$(black squares). The linewidth value δν= 0.032 THz has been chosen as 10% of the linewidth of the narrowest peak to ensure the condition of validity of Equation (8), $\delta \nu \ll ∆\nu_{t}$, for all hole radii. The ideal spectral sensitivity is relatively constant, ranging from 0.24 RIU^−1^ to 0.27 RIU^−1^. The integrated spectral sensitivity for the high-resolution spectrometric readout varies between 0.032 RIU^−1^ and 0.073 RIU^−1^ and is, as expected, significantly smaller than with the ideal readout. The calculated values of $S_{int,HR}$ slightly increase towards a smaller normalized radius, as it is expected for a better match with the very narrow readout linewidth of the considered spectrometer. The same trend is also seen in the traditional $FoM_{\nu }$, as observed previously for narrow linewidth [7,8]. However, the relative increase from 0.53 RIU^−1^ to 4.54 RIU^−1^ is much stronger than that of $S_{int,HR}$. As the latter is related to the physical limit to the sensing precision, this comparison highlights that the traditional $FoM_{\nu }$ largely overestimates the dependence of the imprecision on the linewidth, even for the case of high-resolution readout equipment, and thereby biases the transducer design towards smaller linewidths. In reality, significantly better performance can be reached by co-optimizing the transducer and readout unit, resulting in different transducer designs. We should note that the absolute values of $S_{int,HR}$ and $S_{ideal}$ depend on the choice of ∆ν, and $S_{int,HR}$ additionally depends on δν. This should be taken into account when comparing their values. Their most direct application is comparing the performance of different transducers for a certain readout method.

For the second example, the multilayer structure, the thickness of the silicon layer on a silica substrate is varied from 400 nm to 10 μm. Figure 5b displays three examples of the respective reflectance spectra. As the refractive index contrast on both surfaces remains the same, all transducers feature the same finesse. Therefore, the typical feature width scales from 1.8 THz to 42 THz as the period of the FP fringes changes from 4.3 THz to 108 THz. While the FP fringes extend over all frequencies, the frequency range chosen for this analysis was from $\nu_{min}=277.4$ THz to $\nu_{max}=322.6$ THz. Figure 5d shows the same three FoMs as in (c) for the transducer’s responses to an index change x in the biolayer. The ideal spectral sensitivity $S_{ideal}$ (pink line) is again calculated from Equation (12). The spectral sensitivity $S_{int,HR}$ (green line) is calculated from Equation (8) for the case of a high-resolution spectrometric readout with linewidth δν= 0.177 THz (also 256 frequency channels). For the traditional figure of merit $FoM_{\nu }=S_{\nu }/\Delta \nu_{FWHM}$ (black squares), we use the full-width half-maximum values of the reflection dips in the FP spectrum as values for $\Delta \nu_{FWHM}$ and determine the surface sensitivity $S_{\nu }$ from the frequency shift at the central frequency (300 THz). Also here, the linewidth value δν= 0.177 THz was chosen as 10% of the linewidth of the narrowest dip to ensure the condition of validity of Equation (8), $\delta \nu \ll ∆\nu_{S}$, for all multilayer structures. The integrated spectral sensitivity shows no overall trend in the plotted frequency range, for both the ideal and the high-resolution spectrometric readout. As expected, the integrated spectral sensitivity for the ideal readout (0.0155 RIU^−1^) is about one order of magnitude larger than for the spectrometric readout (about 0.0016 RIU^−1^). Periodic oscillating deviations from the average value are observed, damping out at larger thicknesses, which are related to the reflection maxima moving in and out of the integration range. As the integration range is arbitrarily chosen, these oscillations must be seen as a consequence of this choice and are, therefore, not relevant. (In a practical system, the sensing window would not have sharp boundaries and these oscillations would be smoothed out). Apart from these oscillations, the integrated spectral sensitivity is constant for both ideal readout and spectrometric readout. This is due to the fact that the average values of $\left|\frac{\partial R_{t}\left(\nu \right)}{\partial x}\right|$ and ${\left|\frac{\partial R_{t}\left(\nu \right)}{\partial x}\right|}^{2}$ are independent of the thickness, which was confirmed through simulations. While the traditional $FoM_{\nu }=S_{\nu }/\Delta \nu_{FWHM}$ is not typically used for the analysis of a multipeaked transducer such as a FP, for reference, Figure 5d also reports its value corresponding to the central peak. As expected, it remains approximately constant throughout the considered range of thicknesses, as the decrease in $\Delta \nu_{FWHM}$ for increasing thickness is compensated by the decrease in $S_{\nu }$.

The integrated spectral sensitivity allows comparison of a broader class of transducers with each other, for a given readout scheme. For both transducer types, we have considered a spectrometric readout with 256 readout channels, which defines the ratio of spectral range Δν and linewidth δν. These two forms of spectrometric readout could be realized using the same spectrometer using two different refraction gratings. For the PhC transducer, the integrated spectral sensitivity with this spectrometric readout ranges from 0.33 RIU^−1^ to 0.74 RIU^−1^, over two orders of magnitude larger than for the multilayer structure with 0.0016 RIU^−1^, therefore leading to a Cramér–Rao lower bound over two orders of magnitude lower. This comparison refers to a situation of equal total light source power, implying a spectral power density about 5.4 times smaller for the FP transducer compared to the PhC structure. The $S_{ideal}$ is also over one order of magnitude larger for the PhC ($S_{ideal}\approx 0.26\;$RIU^−1^) than for the multilayer ($S_{ideal}=$ 0.0155 RIU^−1^). This comparison clearly shows that the PhC transducer is a better choice as biosensor, which is consistent with the large body of experimental biosensing literature using PhCs [5]. However, as observed in Figure 5c, this is not due to the narrower linewidths which are typically obtained in PhCs, but rather to the high sensitivity to surface refractive index changes, due to the high field localization. While we do not consider nanoplasmonic transducers explicitly in this work, we expect that even higher integrated spectral sensitivities can be obtained for both ideal and spectrometric readout, if a readout resolution of the same order as the transducer linewidth is chosen, as illustrated in Figure 2. We stress again that striving for narrower transducer linewidth, as motivated by the traditional $FoM_{\nu }$, will, in general, not lead to better sensing performance if the readout is chosen correctly.

It should be noted that the definition of integrated spectral sensitivity is directly related to the physically achievable imprecision in a sensing system, for a fixed total available light source power and noise at the photodetectors, as expressed by the relation to the Cramér–Rao lower bound, $CRLB=\sigma_{I}/(P\frac{e}{h\bar{\nu }}S_{int})$. In a practical situation, other aspects will play a role, such the availability of light sources, low-noise photodetectors and filters in a given spectral range. In any given case, the best possible imprecision can be calculated directly from Equation (3) for different transducers and readout given the spectral range and power density of the light source. Additionally, different spectral properties of the transducer and readout may affect the ability of prediction algorithms (based on fitting or other methods) to actually reach the CRLB. Despite these practical complications, the general definition of the $S_{int}$ in Equation (4) and its expression for ideal (Equation (12)) and high-resolution spectrometric (Equation (8)) readouts provide a simple and physically sound starting point for comparing the potential performance of sensor systems and spectral transducers, respectively.

## 4. Discussion and Conclusions

In conclusion, our analysis has shown that the established figures of merit, which are commonly used to assess the performance of a transducer and optimize its design, are not suited to comparing the physical limits of sensing performance when the readout is considered as part of the design problem. Looking at the fundamental limit to achievable imprecision, we found that the performance of a sensing system based on a spectral transducer is largely influenced by the properties of the readout system. We have shown that commonly used high-resolution readout equipment based on tunable lasers or spectrometers not only has practical drawbacks in terms of cost, robustness and speed, but also limits the achievable imprecision for transducers with a significantly larger linewidth. In fact, for spectrometric readout schemes with closely spaced frequency channels, the best sensing performance is reached if the transducer linewidth and the resolution of the readout unit are of the same order. This contradicts the common assumption that the best sensing performance can be achieved with the highest spectral resolution. We suggest the integrated spectral sensitivity as an alternative figure of merit to compare transducers with different characteristics in their reflection spectra, which consequently need different readout schemes to function optimally. Through a series of simulations, we demonstrate that this figure of merit can drive the design process in different directions compared to the traditional figure of merit, resulting in better sensing performance when combined with an adapted readout. In particular, we show that the transducer linewidth plays a much more limited role in the sensing performance than previously reported [7,8] and generally assumed. While our analysis has been focused on transduction and readout in the spectral domain, the proposed global approach can also be applied to other transduction modalities, e.g., in the spatial or temporal domain, and to multimodal sensing approaches. Our results question the traditional methods of comparing the performance of transducers for sensing, which ignore the impact of the readout, and put forward a different approach for comparisons between different transducer designs and classes of transducers.

## Data Availability

The raw data supporting the conclusions of this article will be made available by the authors upon reasonable request.

## Appendix A

In the case of a readout system based on a tunable laser and a broad photodetector, the laser is sequentially tuned to a set of frequencies $\left\{\nu_{i}\right\}$ within a frequency range between $\nu_{min}$ and $\nu_{max}$. Again, we assume the width of the spectral range $∆\nu =\nu_{max}-\nu_{min}$ to be much smaller than the mean frequency ($∆\nu \ll \bar{\nu }=\frac{\nu_{max}+\nu_{min}}{2}$). The linewidth of the laser is typically much smaller than any spectral feature in the reflection spectrum of the transducer, which allows simplifying the integral (1). If we assume that the photodetector efficiency and the system transmission (both incorporated in a transmittance T) are constant in the spectral interval ∆ν, the photocurrents measured at the different frequencies, $I_{i}=I\left(\nu_{i}\right)$ are given by

(A1) $$I_{i}=\frac{e}{h\bar{\nu }}P{TR}_{t}\left(\nu_{i}\right).$$

Analogously to the case for a broad light source with several readout channels, the measurand of interest, x, influences only the sensor’s reflection spectrum, leading to

(A2) $$\frac{\partial I_{i}}{\partial x}=\frac{e}{h\bar{\nu }}PT{\left.\frac{\partial R_{t}}{\partial x}\right|}_{\nu_{i}}.$$

This expression can be inserted into the general Equation (4) for the CRLB, leading to

(A3) $$CRLB=\frac{\sigma_{I}}{\sqrt{\sum_{i=1}^{n}{\left(\frac{\partial I_{i}}{\partial x}\right)}^{2}}}=\frac{\sigma_{I}}{P\frac{e}{h\bar{\nu }}S_{laser}},$$

where the integrated spectral sensitivity for laser readout, $S_{laser}$, is defined as

(A4) $$S_{laser}\equiv T\sqrt{\sum_{i=1}^{n}{\left({\left.\frac{\partial R_{t}}{\partial x}\right|}_{\nu_{i}}\right)}^{2}}.$$

The comparison of Equation (A3) and Equations (3) and (9) shows that the integrated spectral sensitivity has the same relation to the CRLB as for the broad light source case. Additionally, as expected, $S_{laser}$ as defined in Equation (A4) has the same dependence on the properties of the transducer as $S_{int,HR}$ derived for the case of broad light source and high-resolution spectrometric readout in Equation (9). However, the prefactor is different as the full laser power is used for each spectral measurement, which will typically result in lower imprecisions in the case of the laser readout. Here, care must be taken in comparing different measurement schemes, particularly those involving different numbers of spectral points n. Indeed, for a fixed total measurement time, the integration time for each measurement and, therefore, the current noise $\sigma_{I}$ will depend on n: $\sigma_{I}\propto \frac{1}{\sqrt{T_{1}}}=\frac{\sqrt{n}}{\sqrt{T_{tot}}}$, where $T_{1}\;$is the integration time for each frequency point and $T_{tot}$ is the total measurement time. The CRLB, therefore, becomes independent of n in the limit of large n. It should also be noted that, for a laser readout, the noise may be limited by shot noise and, therefore, scale with the photocurrent.

This separate derivation shows that the integrated spectral sensitivity is also a plausible and useful figure of merit for a laser-based readout system. However, the different optical power P and the noise current $\sigma_{I}$ have to be considered carefully when the integrated spectral sensitivity is used for a comparison between a laser-based readout system and a readout with a spectrally broad light source for a specific transducer. For simplicity, the discussion of the conclusions drawn from the derived figure of merit in the main text is focused on the case of a spectrally broad light source only.

## References

[1] Schasfoort R.B.M. (2017). Handbook of Surface Plasmon Resonance.

[2] Do T., Ho F., Heidecker B., Witte K., Chang L., Lerner L. (2008). A rapid method for determining dynamic binding capacity of resins for the purification of proteins. Protein Expr. Purif..

[3] Cunningham B., Li P., Lin B., Pepper J. (2002). Colorimetric resonant reflection as a direct biochemical assay technique. Sens. Actuators B Chem..

[4] Chen J., Liu B., Zhang H. (2011). Review of fiber Bragg grating sensor technology. Front. Optoelectron. China.

[5] Pitruzzello G., Krauss T.F. (2018). Photonic crystal resonances for sensing and imaging. J. Opt..

[6] Choi I., Choi Y. (2012). Plasmonic Nanosensors: Review and Prospect. IEEE J. Sel. Top. Quantum Electron..

[7] White I.M., Fan X. (2008). On the performance quantification of resonant refractive index sensors. Opt. Express. OE.

[8] Hu J., Sun X., Agarwal A., Kimerling L.C. (2009). Design guidelines for optical resonator biochemical sensors. J. Opt. Soc. Am. B JOSAB.

[9] Sinibaldi A., Danz N., Descrovi E., Munzert P., Schulz U., Sonntag F., Dominici L., Michelotti F. (2012). Direct comparison of the performance of Bloch surface wave and surface plasmon polariton sensors. Sens. Actuators B Chem..

[10] Conteduca D., Arruda G.S., Barth I., Wang Y., Krauss T.F., Martins E.R., Beyond Q. (2022). The Importance of the Resonance Amplitude for Photonic Sensors. ACS Photonics.

[11] Slabý J., Homola J. (2022). Performance of label-free optical biosensors: What is figure of merit (not) telling us?. Biosens. Bioelectron..

[12] Daaboul G.G., Vedula R.S., Ahn S., Lopez C.A., Reddington A., Ozkumur E., Ünlü M.S. (2011). LED-based Interferometric Reflectance Imaging Sensor for quantitative dynamic monitoring of biomolecular interactions. Biosens. Bioelectron..

[13] Triggs G.J., Wang Y., Reardon C.P., Fischer M., Evans G.J.O., Krauss T.F. (2017). Chirped guided-mode resonance biosensor. Optica.

[14] Cano-Velázquez M.S., Buntinx S., Hendriks A.L., Van Klinken A., Li C., Heijnen B.J., Dolci M., Picelli L., Abdelkhalik M.S., Sevo P. (2024). Beyond spectral resolution in optical sensing: Picometer-level precision with multispectral readout. arXiv.

[15] Chen C., Wang J. (2020). Optical biosensors: An exhaustive and comprehensive review. Analyst.

[16] Paulsen M., Jahns S., Gerken M. (2017). Intensity-based readout of resonant-waveguide grating biosensors: Systems and nanostructures. Photonics Nanostruct. Fundam. Appl..

[17] Moiseev L., Unlü M.S., Swan A.K., Goldberg B.B., Cantor C.R. (2006). DNA conformation on surfaces measured by fluorescence self-interference. Proc. Natl. Acad. Sci. USA.

[18] Emsley M.K., Dosunmu O., Unlu M.S. (2002). High-speed resonant-cavity-enhanced silicon photodetectors on reflecting silicon-on-insulator substrates. IEEE Photonics Technol. Lett..

[19] Conteduca D., Barth I., Pitruzzello G., Reardon C.P., Martins E.R., Krauss T.F. (2021). Dielectric nanohole array metasurface for high-resolution near-field sensing and imaging. Nat. Commun..

[20] Kay S.M. (1993). Fundamentals of Statistical Signal Processing, Volume 1: Estimation Theory.

[21] Soyemi O., Eastwood D., Zhang L., Li H., Karunamuni J., Gemperline P., Synowicki R.A., Myrick M.L. (2001). Design and Testing of a Multivariate Optical Element: The First Demonstration of Multivariate Optical Computing for Predictive Spectroscopy. Anal. Chem..

[22] Multivariate Optical Elements (MOEs). https://www.thorlabs.com.

